# β-d-Altrose

**DOI:** 10.1107/S1600536809000397

**Published:** 2009-01-10

**Authors:** Yuji Watanabe, Hiromi Yoshida, Kosei Takeda, Tomohiko Ishi, Shigehiro Kamitori

**Affiliations:** a Division of Structural Biology, Life Science Research Center and Faculty of Medicine, Kagawa University, 1750-1 Ikenobe, Miki-cho, Kita-gun, Kagawa 761-0793, Japan; b Faculty of Engineering, Kagawa University, 2217-20 Hayashi-machi, Takamatsu, Kagawa 761-0396, Japan

## Abstract

The mol­ecule of the title compound, C_6_H_12_O_6_, [systematic name: (2*R*,3*S*,4*R*,5*R*,6*R*)-6-(hydroxy­meth­yl)oxane-2,3,4,5-tetrol] adopts a ^4^ *C*_1_ chair conformation with the anomeric hydroxyl group in the equatorial position. All hydroxyl groups act as donors and acceptors in hydrogen bonding and the mol­ecule is involved in ten inter­molecular O—H⋯O inter­actions [O⋯O = 2.672 (5)–2.776 (4) Å] with eight neighbouring mol­ecules. Two independent O—H⋯O—H⋯ helices extending along the *z* axis are found in this structure.

## Related literature

For the crystal structure of methyl α-d-altrose, see: Gatehouse & Poppleton (1971 ▶).
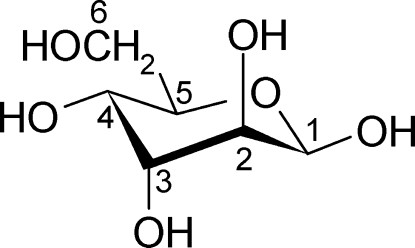


## Experimental

### 

#### Crystal data

C_6_H_12_O_6_
*M* *_r_* = 180.16
Trigonal, 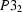

*a* = 7.1749 (13) Å
*c* = 12.7415 (15) Å
*V* = 568.04 (16) Å^3^
*Z* = 3
Cu *K*α radiation
μ = 1.25 mm^−1^
*T* = 293 (2) K
0.30 × 0.30 × 0.30 mm

#### Data collection

Rigaku RAPID2 diffractometer
Absorption correction: none
6207 measured reflections
736 independent reflections
719 reflections with *I* > 2σ(*I*)
*R*_int_ = 0.113

#### Refinement

*R*[*F* ^2^ > 2σ(*F* ^2^)] = 0.046
*wR*(*F* ^2^) = 0.125
*S* = 1.15
736 reflections
109 parameters
1 restraint
H-atom parameters constrained
Δρ_max_ = 0.24 e Å^−3^
Δρ_min_ = −0.24 e Å^−3^

### 

Data collection: *PROCESS-AUTO* (Rigaku, 1998 ▶); cell refinement: *PROCESS-AUTO*; data reduction: *PROCESS-AUTO*; program(s) used to solve structure: *SHELXS97* (Sheldrick, 2008 ▶); program(s) used to refine structure: *SHELXL97* (Sheldrick, 2008 ▶); molecular graphics: *ORTEPIII* (Burnett & Johnson, 1996 ▶) and *ORTEP-3* (Farrugia, 1997 ▶); software used to prepare material for publication: *SHELXL97*.

## Supplementary Material

Crystal structure: contains datablocks global, I. DOI: 10.1107/S1600536809000397/gk2181sup1.cif

Structure factors: contains datablocks I. DOI: 10.1107/S1600536809000397/gk2181Isup2.hkl

Additional supplementary materials:  crystallographic information; 3D view; checkCIF report

## Figures and Tables

**Table 1:** Hydrogen-bond geometry (Å, °)

*D*—H⋯*A*	*D*—H	H⋯*A*	*D*⋯*A*	*D*—H⋯*A*
O1—H*O*1⋯O4^i^	0.82	1.97	2.743 (5)	156
O2—H*O*2⋯O3^ii^	0.82	1.96	2.768 (5)	169
O3—H*O*3⋯O6^iii^	0.82	1.88	2.672 (5)	162
O4—H*O*4⋯O1^iv^	0.82	1.94	2.748 (5)	167
O6—H*O*6⋯O2^v^	0.82	1.96	2.776 (4)	174

Symmetry codes: (i) 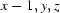; (ii) 
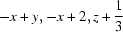
; (iii) 
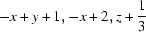
; (iv) 
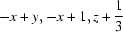
; (v) 
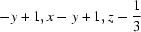
.

**Fig. 1.**
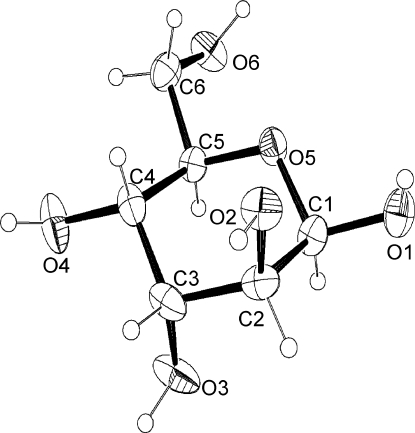
A view of the molecule of β-D-altrose, showing the atom-labelling scheme. Displacement ellipsoids are drawn at the 50% probability level.

**Fig. 2.**
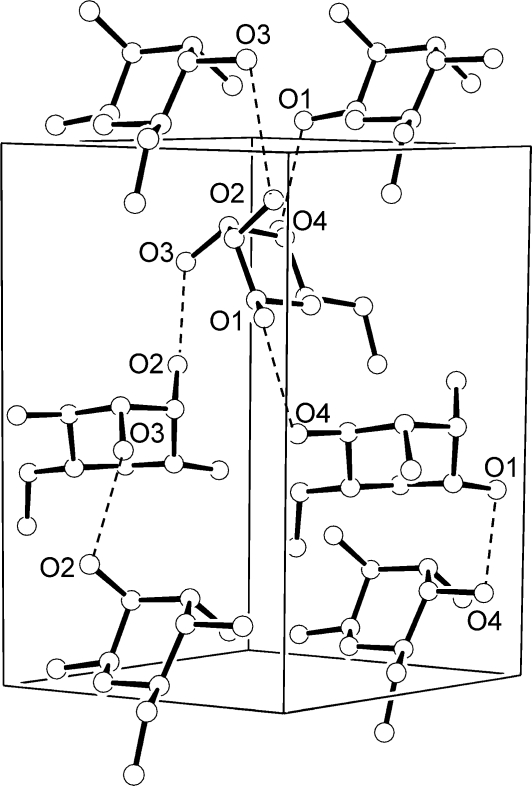
Crystal packing of β-D-altrose, with two helices along the z axis shown as dashed lines.

## supplementary crystallographic information

### Comment

The molecular structure of β-D-altrose is shown in Fig. 1. The aldopyranose ring adopts a ^4^C_1_ chair conformation and the anomer hydroxyl group is in equatorial position pointing to a β-anomer structure. All bond distances and angles between non-hydrogen atoms of β-D-altrose are in the normal range, and torsion angles along C—C and C—O bonds show staggered conformations.

The crystal of β-D-altrose belongs to a trigonal crystal system, space group <it>P</it>3_2_, which is for the first time found in the crystal structure of aldohexoses.

### Experimental

D-Altrose was purchased from Sigma-Aldrich Ltd., Japan. Crystals were prepared by dissolving 20 mg of  D-altrose in distilled water (4 ml). Suitable crystals for X-ray data collection were obtained by slow evaporation of this solution at 293 K.

### Refinement

In the absence of significant anomalous scattering effects, Friedel pairs were averaged. The absolute structure was assigned from the known hand of the starting material. Hydrogen atoms were treated as riding, with C—H distances of 0.97-0.98 Å and O—H distances of 0.82 Å and *U_i__s__o_*(H) = 1.2Ueq(C,O).

### Crystal data

**Table:**

C_6_H_12_O_6_	*D*_x_ = 1.580 Mg m^−^^3^
*M**_r_* = 180.16	Cu *K*α radiation, λ = 1.54178 Å
Trigonal, *P*3_2_	Cell parameters from 2323 reflections
Hall symbol: P 32	θ = 7.2–68.0°
*a* = 7.1749 (13) Å	µ = 1.25 mm^−^^1^
*c* = 12.7415 (15) Å	*T* = 293 K
*V* = 568.04 (16) Å^3^	Block, colorless
*Z* = 3	0.30 × 0.30 × 0.30 mm
*F*(000) = 288	

### Data collection

**Table:**

Rigaku RAPID2 diffractometer	719 reflections with *I* > 2σ(*I*)
Radiation source: fine-focus sealed tube	*R*_int_ = 0.113
graphite	θ_max_ = 71.8°, θ_min_ = 7.1°
ω scans	*h* = −8→8
6207 measured reflections	*k* = −8→8
736 independent reflections	*l* = −15→14

### Refinement

**Table:**

Refinement on *F*^2^	Primary atom site location: structure-invariant direct methods
Least-squares matrix: full	Secondary atom site location: difference Fourier map
*R*[*F*^2^ > 2σ(*F*^2^)] = 0.046	Hydrogen site location: inferred from neighbouring sites
*wR*(*F*^2^) = 0.125	H-atom parameters constrained
*S* = 1.15	*w* = 1/[σ^2^(*F*_o_^2^) + (0.0525*P*)^2^ + 0.4167*P*] where *P* = (*F*_o_^2^ + 2*F*_c_^2^)/3
736 reflections	(Δ/σ)_max_ < 0.001
109 parameters	Δρ_max_ = 0.24 e Å^−^^3^
1 restraint	Δρ_min_ = −0.24 e Å^−^^3^

### Special details

**Table:**

Geometry. All esds (except the esd in the dihedral angle between two l.s. planes) are estimated using the full covariance matrix. The cell esds are taken into account individually in the estimation of esds in distances, angles and torsion angles; correlations between esds in cell parameters are only used when they are defined by crystal symmetry. An approximate (isotropic) treatment of cell esds is used for estimating esds involving l.s. planes.
Refinement. Refinement of *F*^2^ against ALL reflections. The weighted *R*-factor *wR* and goodness of fit *S* are based on *F*^2^, conventional *R*-factors *R* are based on *F*, with *F* set to zero for negative *F*^2^. The threshold expression of *F*^2^ > σ(*F*^2^) is used only for calculating *R*-factors(gt) *etc*. and is not relevant to the choice of reflections for refinement. *R*-factors based on *F*^2^ are statistically about twice as large as those based on *F*, and *R*- factors based on ALL data will be even larger.

### Fractional atomic coordinates and isotropic or equivalent isotropic displacement parameters (Å^2^)

**Table:**

	*x*	*y*	*z*	*U*_iso_*/*U*_eq_	
C1	0.3898 (7)	1.0634 (7)	0.3892 (3)	0.0257 (9)	
H1	0.4984	1.1575	0.3381	0.031*	
C2	0.4763 (7)	1.1433 (7)	0.4977 (4)	0.0299 (10)	
H2	0.5069	1.2918	0.5054	0.036*	
C3	0.6856 (7)	1.1365 (7)	0.5119 (4)	0.0309 (10)	
H3	0.7366	1.1760	0.5843	0.037*	
C4	0.6474 (7)	0.9113 (8)	0.4889 (4)	0.0288 (9)	
H4	0.5474	0.8118	0.5415	0.035*	
C5	0.5476 (6)	0.8366 (7)	0.3805 (3)	0.0253 (8)	
H5	0.6491	0.9295	0.3267	0.030*	
C6	0.4840 (8)	0.6073 (7)	0.3594 (4)	0.0306 (9)	
H6A	0.6000	0.5832	0.3807	0.037*	
H6B	0.3583	0.5139	0.4011	0.037*	
O1	0.1989 (5)	1.0605 (6)	0.3667 (2)	0.0349 (8)	
HO1	0.1136	1.0032	0.4151	0.042*	
O2	0.3161 (5)	1.0090 (5)	0.5728 (3)	0.0309 (7)	
HO2	0.3499	1.0649	0.6309	0.037*	
O3	0.8420 (6)	1.2911 (6)	0.4415 (3)	0.0435 (9)	
HO3	0.9453	1.3817	0.4750	0.052*	
O4	0.8438 (6)	0.9049 (7)	0.4941 (3)	0.0441 (9)	
HO4	0.8688	0.8905	0.5555	0.053*	
O5	0.3537 (5)	0.8494 (5)	0.3754 (3)	0.0267 (7)	
O6	0.4365 (5)	0.5522 (5)	0.2508 (3)	0.0362 (8)	
HO6	0.3057	0.4819	0.2428	0.043*	

### Atomic displacement parameters (Å^2^)

**Table:**

	*U*^11^	*U*^22^	*U*^33^	*U*^12^	*U*^13^	*U*^23^
C1	0.034 (2)	0.028 (2)	0.021 (2)	0.0197 (17)	−0.0044 (17)	−0.0027 (16)
C2	0.037 (2)	0.027 (2)	0.020 (2)	0.0118 (19)	0.0030 (18)	0.0018 (16)
C3	0.027 (2)	0.035 (2)	0.018 (2)	0.0056 (18)	0.0015 (17)	0.0001 (16)
C4	0.025 (2)	0.044 (2)	0.018 (2)	0.0172 (19)	0.0026 (16)	0.0059 (18)
C5	0.026 (2)	0.030 (2)	0.024 (2)	0.0169 (17)	0.0009 (15)	0.0031 (16)
C6	0.038 (2)	0.036 (2)	0.022 (2)	0.021 (2)	0.0038 (18)	0.0017 (18)
O1	0.0384 (17)	0.0461 (18)	0.0308 (18)	0.0291 (15)	0.0000 (13)	−0.0011 (14)
O2	0.0343 (17)	0.0324 (16)	0.0248 (15)	0.0157 (14)	0.0036 (13)	−0.0009 (13)
O3	0.0348 (18)	0.0380 (19)	0.0288 (18)	−0.0034 (14)	0.0043 (15)	0.0018 (15)
O4	0.0302 (17)	0.077 (3)	0.0334 (19)	0.0334 (19)	−0.0011 (14)	0.0063 (18)
O5	0.0262 (15)	0.0278 (15)	0.0283 (15)	0.0152 (13)	−0.0072 (12)	−0.0043 (12)
O6	0.0282 (15)	0.0430 (18)	0.0367 (19)	0.0172 (15)	0.0019 (13)	−0.0121 (15)

### Geometric parameters (Å, °)

**Table:**

C1—O1	1.389 (5)	C4—H4	0.9800
C1—O5	1.435 (5)	C5—O5	1.441 (5)
C1—C2	1.506 (6)	C5—C6	1.495 (6)
C1—H1	0.9800	C5—H5	0.9800
C2—O2	1.435 (5)	C6—O6	1.432 (6)
C2—C3	1.537 (6)	C6—H6A	0.9700
C2—H2	0.9800	C6—H6B	0.9700
C3—O3	1.431 (5)	O1—HO1	0.8199
C3—C4	1.526 (6)	O2—HO2	0.8188
C3—H3	0.9800	O3—HO3	0.8199
C4—O4	1.434 (5)	O4—HO4	0.8206
C4—C5	1.524 (6)	O6—HO6	0.8199
			
O1—C1—O5	108.1 (3)	O4—C4—H4	108.6
O1—C1—C2	114.3 (4)	C5—C4—H4	108.6
O5—C1—C2	109.8 (3)	C3—C4—H4	108.6
O1—C1—H1	108.2	O5—C5—C6	106.8 (3)
O5—C1—H1	108.2	O5—C5—C4	108.5 (3)
C2—C1—H1	108.2	C6—C5—C4	112.5 (3)
O2—C2—C1	108.5 (4)	O5—C5—H5	109.7
O2—C2—C3	111.5 (4)	C6—C5—H5	109.7
C1—C2—C3	108.7 (4)	C4—C5—H5	109.7
O2—C2—H2	109.4	O6—C6—C5	112.2 (4)
C1—C2—H2	109.4	O6—C6—H6A	109.2
C3—C2—H2	109.4	C5—C6—H6A	109.2
O3—C3—C4	110.9 (4)	O6—C6—H6B	109.2
O3—C3—C2	107.5 (4)	C5—C6—H6B	109.2
C4—C3—C2	110.5 (3)	H6A—C6—H6B	107.9
O3—C3—H3	109.3	C1—O1—HO1	109.6
C4—C3—H3	109.3	C2—O2—HO2	109.4
C2—C3—H3	109.3	C3—O3—HO3	109.6
O4—C4—C5	109.0 (4)	C4—O4—HO4	109.1
O4—C4—C3	111.5 (4)	C1—O5—C5	113.6 (3)
C5—C4—C3	110.5 (4)	C6—O6—HO6	109.3
			
O1—C1—C2—O2	58.2 (5)	C2—C3—C4—C5	54.3 (5)
O5—C1—C2—O2	−63.4 (4)	O4—C4—C5—O5	−178.4 (3)
O1—C1—C2—C3	179.6 (3)	C3—C4—C5—O5	−55.6 (4)
O5—C1—C2—C3	58.1 (4)	O4—C4—C5—C6	63.7 (5)
O2—C2—C3—O3	−174.0 (3)	C3—C4—C5—C6	−173.4 (4)
C1—C2—C3—O3	66.4 (4)	O5—C5—C6—O6	74.4 (4)
O2—C2—C3—C4	64.8 (5)	C4—C5—C6—O6	−166.8 (3)
C1—C2—C3—C4	−54.8 (5)	O1—C1—O5—C5	170.9 (3)
O3—C3—C4—O4	56.6 (5)	C2—C1—O5—C5	−63.9 (4)
C2—C3—C4—O4	175.7 (4)	C6—C5—O5—C1	−177.1 (3)
O3—C3—C4—C5	−64.8 (5)	C4—C5—O5—C1	61.5 (4)

### Hydrogen-bond geometry (Å, °)

**Table:**

*D*—H···*A*	*D*—H	H···*A*	*D*···*A*	*D*—H···*A*
O1—HO1···O4^i^	0.82	1.97	2.743 (5)	156
O2—HO2···O3^ii^	0.82	1.96	2.768 (5)	169
O3—HO3···O6^iii^	0.82	1.88	2.672 (5)	162
O4—HO4···O1^iv^	0.82	1.94	2.748 (5)	167
O6—HO6···O2^v^	0.82	1.96	2.776 (4)	174

Symmetry codes: (i) *x*−1, *y*, *z*; (ii) −*x*+*y*, −*x*+2, *z*+1/3; (iii) −*x*+*y*+1, −*x*+2, *z*+1/3; (iv) −*x*+*y*, −*x*+1, *z*+1/3; (v) −*y*+1, *x*−*y*+1, *z*−1/3.

## References

[bb1] Burnett, M. N. & Johnson, C. K. (1996). *ORTEPIII* Report ORNL-6895. Oak Ridge National Laboratory, Tennessee, USA.

[bb2] Farrugia, L. J. (1997). *J. Appl. Cryst.* **30**, 565.

[bb3] Gatehouse, B. M. & Poppleton, B. J. (1971). *Acta Cryst.* B**27**, 871–876.

[bb4] Rigaku (1998). *PROCESS-AUTO* Rigaku Corporation, Tokyo, Japan.

[bb5] Sheldrick, G. M. (2008). *Acta Cryst.* A**64**, 112–122. 10.1107/S0108767307043930 18156677

